# Understanding how adherence goals promote adherence behaviours: a repeated measure observational study with HIV seropositive patients

**DOI:** 10.1186/1471-2458-12-587

**Published:** 2012-08-01

**Authors:** Gareth Jones, Kim Hawkins, Rebecca Mullin, Tamás Nepusz, Declan P Naughton, Paschal Sheeran, Andrea Petróczi

**Affiliations:** 1 Department of Physiotherapy, Guy’s and St Thomas’ NHS Foundation Trust, London, UK; 2 Department of Biological Physics, Eötvös Loránd University, Budapest, Hungary; 3 School of Life Sciences, Kingston University London, London, UK; 4 Department of Psychology, The University of Sheffield, Sheffield, UK

**Keywords:** HIV, Adherence, Health related exercise, Social cognition, Implicit association test, Hair analysis

## Abstract

**Background:**

The extent to which patients follow treatments as prescribed is pivotal to treatment success. An exceptionally high level (> 95%) of HIV medication adherence is required to suppress viral replication and protect the immune system and a similarly high level (> 80%) of adherence has also been suggested in order to benefit from prescribed exercise programmes. However, in clinical practice, adherence to both often falls below the desirable level. This project aims to investigate a wide range of psychological and personality factors that may lead to adherence/non-adherence to medical treatment and exercise programmes.

**Methods:**

HIV positive patients who are referred to the physiotherapist-led 10-week exercise programme as part of the standard care are continuously recruited. Data on social cognitive variables (attitude, intention, subjective norms, self-efficacy, and outcome beliefs) about the goal and specific behaviours, selected personality factors, perceived quality of life, physical activity, self-reported adherence and physical assessment are collected at baseline, at the end of the exercise programme and again 3 months later. The project incorporates objective measures of both exercise (attendance log and improvement in physical measures such as improved fitness level, weight loss, improved circumferential anthropometric measures) and medication adherence (verified by non-invasive hair analysis).

**Discussion:**

The novelty of this project comes from two key aspects, complemented with objective information on exercise and medication adherence. The project assesses beliefs about both the underlying goal such as following prescribed treatment; and about the specific behaviours such as undertaking the exercise or taking the medication, using both implicit and explicit assessments of patients’ beliefs and attitudes. We predict that i) the way people think about the underlying goal of their treatments explains medication and exercise behaviours over and above the effects of the behaviour-specific thinking and ii) the relationship between adherence to exercise and to medical treatment is stronger among those with more favourable views about the goal. Results from this study should identify the key contributing factors to inform subsequent adherence research and afford a more streamlined assessment matrix. The project also aims to inform patient care practices.

****UK Clinical Research Network registration number**:**

UKCRN 7842.

## Background

In the last two decades, owing to the advanced treatment, the long term outlook for HIV-positive patients has considerably improved and now reached 2/3 of the normal life-expectancy at age 20 with medication [1]. The escalating costs of HIV treatment underscore the importance of efficiency in HIV treatment. In addition to the constantly improving medical treatment regime, quality of life of HIV-positive patients has received increased attention. Exercise has been one of the most popular self-care therapies affording many health benefits and improved well-being, assuming low attrition. A body of literature has assessed the beneficial effects of exercise adherence in clinical trials [2-6] but not in home-based intervention [7]. One plausible explanation is that clinical trial studies typically use highly motivated volunteers where adherence is automatically assumed and met. In real life, adherence to exercise and to medical treatment may fall well below desired levels and hinder the effectiveness of such programmes [8].

Exercise is known to be a beneficial auxiliary treatment in many chronic conditions [9]. Studies investigating adherence to exercise in self-managed settings have focused on conditions such as heart failure, injury, rheumatoid arthritis, low back pain or cystic fibrosis [10-12]. Despite the fact that adherence has been identified as a major barrier to benefits from exercise-based treatments [13,14], research among HIV patients are generally focussed on adherence to medications. The number of studies reporting the influence of adherence to exercise among HIV patients is limited. Contrary to published controlled trials showing the positive effect of exercise and reports which indicate that 25% of HIV patients failed to meet daily recommended physical activity guidelines [15,16], an observational study suggested that adherence in the clinical setting is rather low with only half of the referred patients choosing to participate and adhere to the prescribed and physiotherapist-led exercise programme [17]. Apart from the missed benefits from exercise, Kyser et al [18] also found that patients who did not engage in any aerobic exercise in the last 30 days were more likely to be non-adherent to medication.

To date, there is a hiatus in studies investigating the psychosocial determinant of adherence and non-adherence to physiotherapist-led exercise programmes in HIV patients, or exploring the relationship and moderating factors between adherence to medical treatment and prescribed exercise. Furthermore, the relationship between cognitive factors of adherence to medical treatment regimes and exercise has not been investigated.

Understanding factors that influence volitional behaviour has been in the centre of adherence research. Generally, HIV patients indicate a strong intention to take their medications as prescribed, but despite this their observed or self-reported adherence often falls below the optimal level [19]. In relation to adherence to treatment regimes among HIV patients, Munro et al [20] reviewed 6 behavioural models applied to promoting adherence in a limited number of studies but found little evidence for effectiveness. Studies in the last 5 years appear to be reiterating the same conclusion with some finding the Theory of Planned Behaviour (TPB) [21] instrumental to enhance adherence [22] whereas others found that only a small fraction of the variance in intention to adhere to antiretroviral therapy was explained by the classic TPB predictor set of attitude, norms and perceived control [23]. Self-regulatory processes were found to mediate between intention and adherence [24]. Furthermore, outcome beliefs about the medication have been found to be an important factor in determining adherence [25,26].

Attempts have been made in the past to identify ‘high-risk’ patients for non-adherence, relying on relatively stable predictors such as personality or using predictors that are beyond the patients’ control, such as barriers to care or medication, complexity of treatment, costs of medication or required co-payment [27,28]. In fact, in HIV positive individuals the motivating and deterring factors for adherence to medical treatment yield a rather complex picture [29]. As in most self-regulated behaviour, personality characteristics influence the self-regulatory process in HIV adherence. Adherent patients are characterised by feeling in control, are empowered and showing proactiveness, as opposed to non-adherent patients’ perception of being a ‘victim’ of the HIV treatment regime, passiveness and disempowerment [30]. In clinical practice, low self-efficacy or depression has been thought to be related to non-adherence to medical treatment and/or rehabilitation [31-35]. Adherent patients perceived their quality of life to be higher [36] which is in line with previous findings showing that those with better well-being are more likely to show interest in further improvements [37]. The role of socioeconomic factors in HIV treatment adherence is ambiguous [38] but gender [39] and ethnic [18,40,41] differences were observed.

### Scientific justification and theoretical framework

**Figure 1:**
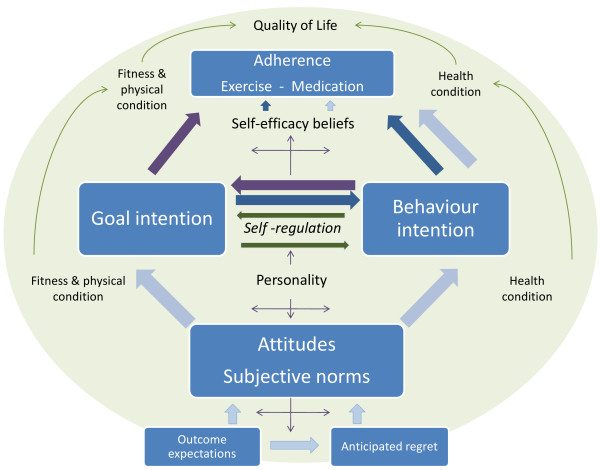
**Conceptual framework of medication and exercise adherence.** Light blue arrows represent the behavioural intention pathway (TPB). Goal intention models: dark blue arrows denote a pathway where goal intention is an antecedent; dark burgundy arrows show a pathway where goal intention is a moderator of the behavioural intention.

The novelty of this project comes from the theoretical framework applied and the measurements utilised in data collection. From the theoretical point of view, the key aspect of the project is that it is assessing cognitions about both the underlying goal of following prescribed treatment and about the specific behaviours such as taking medication as prescribed and exercise (Figure1). From the methodological aspects, the novel elements are the consideration of both implicit and explicit cognitions, along with using objective measures of both exercise (attendance and clinically meaningful improvement in physical measures, such as improved fitness level, weight loss, improved circumferential anthropometric measures, improved one repetition maximum strength measures) and medication adherence (hair analysis).

The TPB, along with other social cognitive models [42] and specifically in relation to health actions [43], assumes that any given behaviour is a functional outcome of the attitude – intention – behaviour sequence, which makes the model suitable for understanding and predicting volitional health-preserving or improving behaviour. The efficacy of the TPB in relation to health-related behaviour is dependent on the particular behaviour (*i.e.* adhering to health improvement or engaging in risky activities), age, time-frame and measurements [44]. Improvement made in order to increase the predictive power of the TPB model is abundant, including descriptive norms [45], subjective norms [46], self-identity [47], self-efficacy and locus of control [48], behavioural control [49], anticipated regret [50,51], desires and emotions [52], moral norms and anticipated affect [53], social cognition properties [54], prototypes and willingness [55], conscientiousness [56] and goals and its properties [57]. The variations of the augmented TPB models acknowledges the complexity of factors underlying the ultimate behavioural choice but nevertheless assume that the behaviour is predicted by some combination of social cognitive factors about the behaviour. Whilst each added variable incrementally increases the predictive power of the TPB model, it inadvertently introduces some unexplained variances with each predictor, thus the price to be paid for the improvement manifests in complexity. Meta-analyses have shown that beliefs and attitudes in the TPB model predict 39% of the behavioural intention and 27% of the actual behavior, with notably stronger prediction when behavior was based on self-reports [58]. In order to close the gap between behavioural intention and actual behaviour, Gollwitzer and Sheeran [59] proposed the addition of implementation intention.

Approaching behaviour from a different angle, the Control Theory [60] proposes that behavioural choices are driven by goal pursuits, where desired goals induce intentions but also serves as reference values against which the progress of achieving the goals are constantly monitored and adjusted if necessary. The allowance for incremental approaches to the desired goals connects goal theories of behaviour [57] and implementation intentions [59] via self-regulation by which people control their feelings and impulses to ensure behavioural outcomes that are perceived to be desirable. Self-regulation is a complex process that includes long-term perspectives in constant monitoring attainments [60] as well as resisting temptation with the view of pursuing long term goals [61]. The self-regulatory effect is thought to rely on a limited energy resource that can be temporarily depleted, hence exerting effects on other behavioural choices made simultaneously or shortly after [62].

Investigations of the role of self-regulation in long-term medication adherence [63] revealed that self-regulation not only accounted for the largest explained variance in the self-determination theory-based model but also was the only variable that showed significant correlation with both self-reported adherence to prescribed medication and pill-count. Self-managed care based on a self-regulation framework has been shown to be effective in health-maintenance among people with chronic health conditions including improvement in condition, along with better physical and psychosocial functioning [64]. Other examples for interventions based on self-regulation theory being successful are related to reducing risk of coronary heart disease [65], reducing depression among people suffering from rheumatoid arthritis [66] and end-stage renal disease [67].

Self-regulation is present in peoples’ daily lives because discrepancies between expectations and reality in situations people encounter are inevitable. Successful adaptive functioning is based on effective self-regulation by which people control their impulses, thoughts, feelings and adjust their behaviour to close or minimize the gap between their expectations and present experiences. The information processing models of self-regulation focuses on the process of constant comparison and feedback between the current and the desired, informing the necessary adjustment required to the behaviour [68]. However, the way people carry out this task is a reflection of their individual dispositions. General personality dimensions and specific self-referent personality traits are thought to be inseparably intertwined with self-regulation [69,70]. General personality dimensions such as conscientiousness [71], impulsiveness vs. constraint [72] and specific self-referent traits such as self-efficacy and self-esteem [73] are key components of the self-regulative process.

Every step of the self-regulative process, which can manifest in active initiation and maintenance of a goal-oriented and effortful activity or inhibition of impulsive behaviour that could jeopardize reaching the desired state, is influenced by the personality factors. Individual differences determine people’s dispositional capacity for self-regulation, as well as affecting the dynamics of the regulatory processes [69]. Stable individual differences determine the self-regulatory strategies individuals automatically employ [74]. Personality traits affect how ambitious the goals are; how discrepancies are viewed; and what self-regulatory strategies are employed. Personality also influences whether people exhibit flexibility in behaviour to achieve the desired goal or increase effort in following the behavioural path they set themselves on, initially assuming that the discrepancy between the desired and current state is to be bridged by trying harder or one is motivated to find alternative ways to reach the desired goals. Integrating trait and process variables self-regulation offers a more comprehensive account of self-regulation [75].

Demographic factors such as gender and age moderate the effect of personality factors on goal setting, self-regulation and behavioural antecedents. Although personality trait changes occur most dominantly in young adulthood (20-40 years of age), they gradually change at a very slow pace over the lifespan: self-control generally increases with age [76-78], along with conscientiousness [79], self confidence and emotional stability [78]. Gender differences are small relative to individual variation within genders and broadly conform with gender stereotypes of males being more assertive than females who perceive themselves higher than males on agreeableness, warmth and neuroticism while being open to feelings, rather than ideas [80] but no difference exists in impulsiveness, locus of control or orderliness [81]. Males possess slightly higher general self-esteem compared to females [82]. On the contrary, ethnicity within the same culture does not seem to exert a significant influence on behaviour but rather culture is seen as aggregate personality profiles [83] that manifest in typical behaviour of most, but not all, people in a given country [84]. Although cultural differences in broad personality traits have been consistently shown in cross-cultural studies [85], within culture differences in personality traits by ethnicity were found to be generally negligible [86].

The integration of the relevant goal-intention-behaviour theories is depicted in Figure1. It shows a conceptual map that underpins the current investigation in adherence to prescribed exercise and treatment regime among HIV positive patients. Light blue arrows show the behavioural intention model based on TPB. Arrows coloured in dark blue and dark burgundy lay out the path for the goal intention model, where explicit and implicit goal intentions are either an antecedent (Goal intention → Behavioural intention → Adherence, shown in dark blue) or a moderator (Behavioural intention → Goal intention → Adherence, shown in dark burgundy) of the behavioural intention. In the latter case, we expect the strength of the path between behavioural intention and adherence being diminished or weakened by the inclusion of goal intention between behavioural intention and adherence [87].

A person’s ability to follow a long-term interest via self-regulation plays a vital role in medical treatment, particularly in conditions that require prolonged health management (e.g. chronic conditions or HIV). Although many segments of self-regulation appear to be in the unconscious and automatic domain, health improvement or maintenance requires conscious, effortful and goal-oriented self-regulatory behaviour. That is not to say that unconsciously held motivators do not play an important role in consciously self-regulated behaviour. Dual process models [88] suggest the existence of a two-tier system where the higher-order system is responsible for reflective and planned behaviour whilst the lower-order system responds to associative cues at the moment [89].

Automatic processes occur without intention or awareness whereas controlled processes are based on intentional effort to either seek information in order to choose the appropriate action or to inhibit automatic processes in order to execute the selected action. Clearly, implicit automatic and explicitly controlled processes are qualitatively different entities that independently contribute to regulating human behaviour [90,91]. Research in implicit social cognition provides ample evidence that thought processes outside the conscious domain influence behaviour and that the processes under conscious control constitute only a small fraction compared to what is present in decisions about behaviour [92]. The distinction between automatic implicit and self-controlled explicit processes has been evidenced in motivation [93], attitudes, stereotypes, self-esteem and self-concept [94,95] with considerable individual differences in awareness of and ability to control automatic processes [92,96] being evidenced. Despite this, most policy development narrow-mindedly assumes that behavioural choices are exclusively under the controlled processes when devising preventive measures and interventions to encourage the desirable behaviour [97]. In order to improve the effectiveness of such measures, social policies aiming to behavioural change should incorporate assessment of both implicit and explicit processes [97].

Therefore for the current project it is proposed that explicit assessment is complemented with implicit associations [98] where appropriate. It is known that individuals may be biased in how they see themselves and this bias is often not recognised by the individual [99]. Self-reported, explicit assessments of individual differences are particularly prone to distortion, which may or may not be deliberate. A current research stream in psychology focusing on the uncontrolled, unconscious processes [95] provides a promising avenue to compliment the explicit assessments. Based on the widespread definition of attitude as a relatively stable tendency to evaluate an attitude object with some degree of like or dislike, implicit attitudes are defined as associative processes reflecting this tendency [100]. Associative evaluations are the result of stimulus driven, uncontrolled, unintentional, goal independent or unconscious processes [101] and as such, they do not require respondents to be aware of these attitudes and may be free of self-presentation distortion in some key cognitions such as conscienciousness [102]. Recent research has shown that implicit association adds to the predictive power of explicit attitude measures [103] via one of eight possible mechanisms [104]. Based on the assumption that implicit associations either predict or moderate the relationship between antecedents and actual behaviour, Implicit Association Tests (IAT) have been applied to understand consumer behaviour [105-107], job satisfaction among nurses caring for drug addicts [108], doping behaviour [109] academic performance [110], suicide attempts [111,112], addiction [113], dietary change [114], dental hygene [115] or HIV-risk sexual behaviour [116], illustrating the widespread use of the IAT concept. In the current project, using the Brief IAT (BIAT) concept [117], implicit measures include self-efficacy, attitude and norms. Implicit tests use exactly-matched content to explicit measures ensuring that both explicit and implicit measures are equally and directly related to the concept [118,119].

With adherence constructs being the vital outcome measures, it is crucially important to have reliable information on them. Whilst exercise adherence can be easily measured by using the attendance log but adherence to drug treatment is less straightforward [120]. As an objective alternative (at least for research purposes), measuring the levels of anti-HIV drugs in patients' hair has been proposed [121,122]. Methodologies have already been developed for detecting the presence of nevirapine [123], efavirenz, lopinavir and ritonavir [124] and lopinavir/ritonavir and atazanavir in hair [125] using liquid chromatography coupled with tandem mass spectrometry (LC-MS/MS). In this project, hair analysis is planned to test for the presence of the key anti-viral drugs, including new method development for tenofovir and abacavir in hair, to verify self-reports on adherence or non-adherence. As indirect outcome measures, clinically meaningful improvement [126,127] is expected in fitness, circumference measures and perceived quality of life.

### Research question

Although a high level of adherence is required to benefit from prescribed medication and exercise programmes, in clinical practice adherence occurs on a continuum and often falls below the desirable level. Therefore, the primary objective of this project is to investigate psychological and socio-economical factors that may lead to adherence/non-adherence to medical treatment and exercise programmes, with the view of identifying the key contributing factors to inform patient care practices and subsequent adherence research. In addition, the project explores the extent to which explicit and implicit cognitions about the underlying goal predict variance in medication and exercise adherence. Specifically, we predict that cognitions about the underlying goal explain variance in medication and exercise behaviours and in the relationship between adherence to both exercise and adherence to medical treatment over and above the effects of the behaviour specific cognitions (Figure1). The goal intention is an antecedent or a moderator of the behavioural intention.

#### *Hypothesis*

We hypothesise that the way people think about the underlying goal of their treatments explains medication and exercise behaviours over and above the effects of the behaviour-specific thinking. We also predict that the relationship between adherence to exercise and to medical treatment is stronger among those with more favourable views about the goal.

Specifically, based on models predicting health behaviour, we expect that

1. explicit and implicit cognitions (e.g. attitude, subjective norms, etc.) *about the behaviour* predicts variance in medication and exercise behaviours;

2. explicit and implicit cognitions (e.g. attitude, subjective norms, etc.) *about the underlying goal* explains (predicts variance in) medication and exercise behaviours over and above the effects of the behaviour-specific cognitions;

3. personality characteristics have a modifying effect on these predictions;

4. there is a positive relationship between adherence to exercise and adherence to medical treatment; and

5. the relationship between the two is stronger among those with more favourable cognitions about the goal.

## Method

### *Setting and participants*

**Figure 2:**
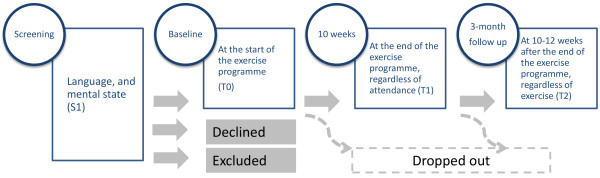
**Study design diagram.** Screening for eligibility (S1), followed by a baseline (T0) and two repeated measures (T1 & T2).

The study utilises a repeated measure design with baseline and two follow-up measures (Figure2). The physiotherapist-led exercise intervention takes place between the baseline and the first follow-up measure. The second follow up measure serves as a checkpoint whether exercise (in case adherence was good during the physiotherapist-led weeks) has been maintained, and if there is any change in the underlying cognitive processes of exercise behaviour and goals. The testing protocols are very similar at T0, T1 and T2 with a minor modification which only manifests in phrasing the question to reflect the actual situation, pre-post treatment and follow up. Variables of the testing protocol are listed in the Additional file 1: Variables and measures.

Data are collected among HIV seropositive patients attending the outpatient clinic at Guy’s and St Thomas’ Hospital London. Willing participants are assessed according to the set criteria for inclusion and exclusion.

Inclusion criteria are: diagnosed as HIV positive, physician assessed and referred to the 10 week gym programme at Guy’s and St Thomas’ Hospital, age between 18-55 and able to give informed consent. Default by inclusion, patients who are medically (physically and/or mentally) unstable are excluded from the study. Further exclusion criteria include those who have attended the programme within the last 3 months; patient with insufficient knowledge of English language to perform the computerised test and/or complete the questionnaire as assessed by the pre-screening questionnaire and inability to provide hair samples owing to lack of hair or having hair with aggressive chemical treatment such as bleaching or perming. Patients with cognitive impairment sufficient to limit ability to perform the computerised test and/or complete the questionnaire are also excluded. Cognitive status is assessed using the Mini Mental State Examination (MMSE) test with a cut off score of 27, following the new guideline [128].

### *Sample size*

The target sample size for recruitment is n = 60 to allow maximum 35% attrition thus reaching the final sample size of 40 with completed datasets. The total sample size consists of two qualitatively different groups: those with good adherence to exercise programme and those with poor adherence/drop out/nonattendance. Constant effort is made to create groups with equal sample sizes.

Sample size calculation was based on the results from a previous study [17]. Finding statistically significant difference/relationship with medium effect size (d = 0.3) at alpha error probability = 0.05, 1 beta error probability = 0.8, two tail analysis for the FAHI Quality of Life (QL) measure [129] between two groups (independent sample): n = 44 (combined sample); FAHI QL measure change in the adherent group (repeated measure): n = 10 (combined sample); and fitness index change in the adherent group (repeated measure): n = 74 (combined sample). The difference in fitness baseline measure between adherent and non-adherent patients was very small.

Information on effect sizes for other measures is not yet available. Using the definition of 'clinically meaningful change', we assume medium effect size. However, a secondary aim of this study is to establish thresholds for clinically meaningful change in the key outcome variables (CD4 count, quality of life, fitness and physical measures). The present study will provide invaluable information for future studies regarding the minimum sample size required to detect clinically significant change (*i.e.* + 20 metres in the 6-minute walk test, + 0.5 SD achieved on the FAHI QL questionnaire) and statistically significant differences.

### *Recruitment of patients*

Eligible participants are continuously recruited by the physiotherapists at St Thomas' Hospital as they are identified by their treating physicians as requiring referral to the 10-week exercise programme as part of their standard care. In addition, posters are placed in waiting rooms informing patients about the study and calling for participants. Finally, the research group are actively involved in disseminating up to date information about the project at specialised interest group meetings and conferences.

### *Data collection*

Each eligible participant is tested three times. The initial assessment is conducted when the patient is referred to the exercise programme, has volunteered to participate, and is eligible for the study (T0). The duration of the physiotherapist-led exercise programme is 10 weeks, comprising of two, 1-hour sessions per week. Participants are tested again at the end of the exercise programme (T1) and again in approximately 10-12 weeks (T2) during which time patients are assumed to maintain their exercise programme individually. All participants are asked to attend the testing sessions, regardless of their level of adherence to exercise. Participants are compensated for their time by gift vouchers (value of £75.00 in total) after each session.

The recruitment is staggered and continuous. The total timeframe for each patient involved in three testing sessions is 25 weeks, including post testing and allowing for delays owing to personal circumstances. Each testing session will last for approximately 90 minutes with short breaks between the implicit assessment test blocks or as required during the completion of the questionnaire and physical testing. To avoid cognitive fatigue, computerised and paper psychological tests are interspersed between the elements of the physical tests.

Participants are asked to donate a hair sample at T2. The hair sample (a diameter of 3 to 4 mms, approximately 50 hairs), 3-4 cm in length and preferably untreated is taken by the attending physiotherapist cutting near the skin surface using a pair of blunt edge scissors in appropriate privacy to observe local patient dignity standards. Depending on hairstyle, if possible, cuts are taken from different locations of the head to avoid bald patches. Hair samples are catalogued by the ID code and stored in a paper envelope tested for the presence of selected Combination Antiretroviral Therapy (cART) drugs and used to verify adherence to medical treatment.

Data on individual patients’ medical record, physical assessment, attendance, psychological tests and hair sampling are linked via numerical codes. Coding is undertaken by the physiotherapy researchers. Other researchers in this project only have access to the coded (hence anonymous) data set. Data collection and analysis are completely separated to negate any researcher bias. Owing to the staggered start-finish of the data collection, the full dataset is not analysed until the data collection is complete.

To meet the objectives, this project is designed to screen a wide range of variables that may contribute to adherence/non-adherence to medical treatment and exercise programmes in HIV positive patients with the view of selecting key variables and establishing testing protocols to inform a subsequent, large scale investigation/intervention study. These variables can be categorised as i) input variables: physical assessment, psychological assessment, medical information, demographics; and ii) outcome variables: adherence to treatment, verified by objective information (drug analysis and attendance log).

### *Pilot testing before finalising the protocol*

A male patient from the exercise programme was involved in reviewing the research protocol, Information sheet for Participants, and Consent form. He provided valuable feedback to the research team and amendments were made throughout to incorporate his comments. The computerised psychological assessment and self-report questionnaire was also tested with two volunteers prior to the data collection. Volunteers were asked to complete the test in the presence of the physiotherapist and 'think loud' (*i.e.* voicing their thought processes). Data collected during this exercise are excluded from the final dataset. The main goal of this exercise was to finalise a testing protocol and the data recording system with as little ambiguity as possible. Patients whom assisted the research in these capacities are excluded from the study.

### *Variables*

Variables for this study were selected from a wide range of factors known from previous research to influence adherence. To capture reasons for adherence/non-adherence beyond verbal declarations, parallel measures of explicit cognitions with implicit associations is planned. This approach will allow us to capture cognitions in situations where participants are i) unaware of what is being measured or ii) have no conscious access to the assessed property or iii) have no control over the measurement outcome. Hair analysis is proposed as an objective measure of adherence and is used to verify self-declared adherence.

#### *Implicit associations*

Implicit measures of attitude, self-efficacy and intention are modified from the BIAT [117]. The BIAT consists of 2 trial blocks with 4 categories, 4 exemplars in each. Contrary to the classic IAT, the BIAT only focuses on 2 of each block’s 4 categories.

**Figure 3:**
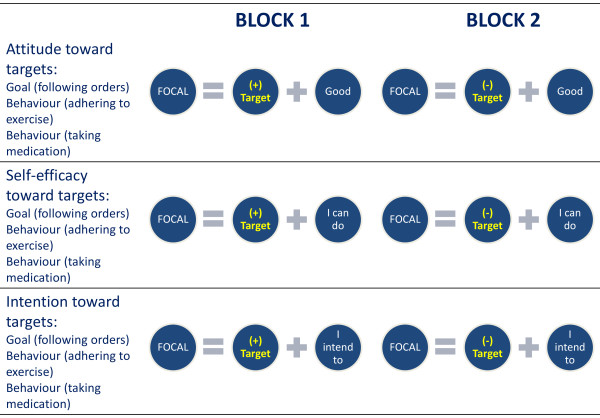
**Setup of the Brief Implicit Association Tests.** Implicit measures of attitude, self-efficacy and intention toward the goal and two behaviours.

For the purpose of this project, the BIAT has been adapted to adherence to i) exercise and ii) medical treatment. Adherence is defined as completing all sessions and taking all medication as prescribed. The adherence category (expressed as frequency) is combined with i) attitude, ii) self-efficacy and iii) behavioural intention measures (Figure3). Each of the nine tests consists of 48 trials in 2 blocks where each block contains 24 trials (8 practice followed by 16 trials). Order of the blocks within each test is counterbalanced; order of the target and the order of the tests are randomised with a short break between every set of 3 IATs, interspersed with physical assessments. Stimuli for the 9 tests are presented in Table1, each appearing 4 times in the trials and each appearing twice for the 8 practice trials. Text in italics indicates the non-focal category. The instructions for each test are presented in Table2.

**Table 1:** Stimuli of the adapted Brief Implicit Association Tests (italics denote the non-focal category)

			**Category**	**Stimuli**
Target	Goal		Following each of my doctor’s orders	all, every, entire, total
			Not following each of my doctor’s orders	none, few, nil, never
	Behaviour		Attending each of my exercise sessions	all, every, entire, total
			Not attending each of my exercise sessions	none, few, nil, never
	Behaviour		Taking each of my medications	all, every, entire, total
			Not taking each of my medications	none, few, nil, never
Attribute	Attitude	(+)	Good	worthwhile, important, pleasant, nice
		*(-)*	*Bad*	*pointless, unimportant, unpleasant, nasty*
	Self-efficacy	(+)	I can do	easy, undemanding, simple, childsplay
		*(-)*	*I cannot do*	*difficult, demanding, hard, tricky*
	Intention	(+)	I intend to	planned, expected, wanted, decided
		*(-)*	*I have no intention*	*unplanned, unexpected, unwanted, undecided*

**Table 2:** Instructions for the modified Brief Implicit Association Tests

**Block**	**Instruction for focal key**	**Instruction for non-focal key**
1	Press ‘I’ for focal concept 1 OR (+) words	Press ‘E’ for anything else [focal concept 2 OR *(-) words*]
2	Press ‘I’ for focal concept 2 OR (+) words	Press ‘E’ for anything else [focal concept 1 OR *(-) words*]

The implicit assessment tests are custom made and programmed under Linux operation system on a laptop computer (Samsung P500, Intel Core 2 duo, 15.4" WXGA screen and full size QWERTY keyboard with individually mounted keys). For security reasons and to prevent interference, all other software has been removed from the laptop.

#### *Self-reported explicit variables*

Explicit measures of attitude contained the exact phrases used in the IAT as stimuli and rated on a 6-point Likert-type scale. Statements for the goal attitudes “*Following each of my doctor’s orders is”,* medication adherence *“Taking each of my medication is*…” and *“Attending each of my exercise sessions is…”* are followed by *worthwhile/important/pleasant/nice/unimportant/pointless/nasty/unpleasant”*, giving 3 sets of 8 statements. Reversed scoring is used for statements containing ‘negative’ words.

Perceived quality of life and overall health in the screening questionnaire were captured by the answers to the questions “*Would you say your overall quality of life is…*” and *‘’Would you say your overall health is…”* on a 7-point scale ranging from excellent through very good, good, fair, poor, very poor to unbearable. The single question self-assessment is based on a previous study demonstrating that a single question self assessment regarding one’s health is a reliable indicator of the general health [130].

Quality of life was assessed in using the revised Functional Assessment of Human Immunodeficiency Virus Infection (FAHI) quality of life instrument [129], measuring 5 facets of well-being: physical (13 items), emotional and living with HIV (10 items), functional and global (13 items), social (8 items) and cognitive functioning (6 items).

Validated scales are used to assess conscientiousness [71], need for cognition [131] and social desirability [132]. Activity level during the study is self-reported at each measurement point using the International Physical Activity Questionnaire [133].

The strength of the combination of motivational forces influencing a behaviour in question is captured in behavioural intention [21], accounting some 30 percent of the variances in behaviour [134]. The questions assessing HIV treatment related intentions are “*I will follow each of my doctor’s orders*”, “*I will take each of my HIV medications*” and “*I will attend each of my exercise sessions / I will continue with exercise*” for goal intention and behavioural intentions to be adherent to medication and exercise regimes, respectively.

Subjective norms are assessed with three questions: “*People who are important to me think that I should.”* i) “*follow doctor’s prescription*” (goal), ii) “*take all my HIV medications*” (adherence to medication) and iii) “*attend for every exercise session / continue with exercise*” (adherence to exercise).

Anticipated regret about non-adherence toward medication and exercise are measured with a single statement each, rated on a 6-point Likert-type scale: “*If I did not take my prescribed medication, I would feel regretful*” and “*If I did not do my prescribed exercises, I would regret*”.

Self efficacy beliefs were measured with three statements rated on a 6-point scale ranging from definitely no to definitely yes. The statements started with “*I am confident that…”* and followed by *“I could take each of my HIV medications”*, “*I will be able to manage my illness”,* and *“I can continue with my exercise”*.

Outcome expectations are assessed by six questions answered on the 5-point scale ranging between not sure at all and very sure. All questions are preceded by ”*How sure are you that…”* with medication and exercise being asked separately. The questions are: “*the medication/exercise will have positive effect on your health”, “the exercise will make you feel better”, “taking your medication as prescribed is necessary to manage your illness to expand your life”, “doing the prescribed exercises will have a long-term benefit on your health and quality of life”, “if you do not take your medication exactly as instructed, your illness will get worse”.*

Self-reported adherence to medication was assessed by a direct question *“When was the last time you missed taking any of your medication?”*, with closed answer options of *“within the past week”*, “1-2 weeks ago”, *“2-4 weeks ago”*, *“1-2 months ago”*, *“more than 2 months ago”* and *“never skip medication”*.

Reasons for non-adherence, if present, to medication and exercise were investigated using the ACTG Baseline Questionnaire [135], with the questionnaire adapted to adherence to exercise as well. Exercise non-adherence related reasons were informed by the informal feedback patients offered to the physiotherapist in the preceding years prior to this study. Data from the exercise non-adherence questions will be tested to ascertain if they form a valid and reliable scale measuring barriers toward exercise adherence in this special population, and the scale will be further developed if promising scale properties are detected in the present sample. The items are presented in Additional file 2: Reasons for non-adherence.

The questionnaires used at the three measurement points are identical apart from minor modification to phrasing the question in order to reflect the actual situation. For example, *“attending each of my exercise session”* in T1 are replaced by *“continue with my exercise”* in T2 and T3.

#### *Objective measures*

The project incorporates objective measures of both exercise and medication adherence. Exercise adherence is assessed via attendance logs during the first phase and self-managed exercise diary during the second phase. Clinically meaningful improvement in fitness and physical indicators (*i.e.* improved fitness level, weight loss, improved circumferential anthropometric measures) also serve as a proxy measure for adherence.

Medical adherence is assessed via self-reports corroborated by non-invasive hair analysis, using 50 mg of cut hair samples. Methods are being developed in house for two HIV drugs, tenofovir and abacavir, as one of the two presents in all treatment combination for this sample. The two drugs are quantified using Liquid Chromatography-Tandem Mass Spectrometry (LC-MS/MS) following extraction from the hair samples. Owing to the limitations of the method, hair analysis provides a crude estimation of adherence and thus is mainly used to flag up individuals with total absence of the drug in their hair. Quantified drug results in hair are compared to the anonymised information on medical treatment.

### *Statistical analysis*

Treating missing data will be decided upon examining the pattern (if any) of the missing data. A missing value may be replaced if necessary, using the average of the closest 4 neighbours (two below and two above), ranking participants by the most similar measure as indicated by the highest correlation between the two measures. Scale reliability is tested by calculating internal consistency coefficients (Cronbach alphas or in case of dichotomous answer mode, Kuder-Richardson formula). Implicit association reliability is calculated using the split-half method. IAT effect is calculated as the difference in average response times between the two focal test blocks, and individually divided by the variance to derive the D-scores [136,137]. To identify factors influencing adherence/non-adherence, discriminant function analysis are used. Using exercise adherence as a continuous measure (frequency from the attendance log) will also allow investigating factors that explain the variance in adherence the most. The relationship between the two types of adherence is tested using chi-square analysis, whereas correlation is used to test relationship between variables measured on a continuous scale. Building upon strong correlations, the adherent/non-adherent behaviour can also be represented by drawing and testing a structural equation model. Standard statistical analysis will be performed using SPSS 19.0, SAS 8.0 and R software packages. Behavioural models will be tested with structural equation modelling, using AMOS 6.0. Achieved statistical power will be estimated with G*Power 3.0.1 [138].

### Project management

#### *Quality control*

Owing to the relatively high cognitive demand of the research protocol (series of IATs and a long questionnaire), volunteering participants are assessed for the cognitive ability and level of English to ensure high quality data. Recruitment is conducted via the standard care to create a 'real life' scenario and avoid motivation bias by recruiting volunteers. The study is designed to investigate the reasons for adherence and non-adherence in every day clinical settings, where the research component is limited to the psychosocial information which is equally important from adherent and non-adherent patients.

#### *Compliance*

Participation in the study is entirely voluntary and independent of their participation of the prescribed exercise programme. All participants are asked to attend the three testing sessions, regardless of their level of adherence to the exercise programme. A £20 shopping voucher (redeemable at a major supermarket) is offered for attending each of the first two testing sessions followed by a £35 shopping voucher for attending the third testing session, subject to attending the previous two.

#### *Data storage and confidentiality*

Confidentiality is maintained throughout the study. Physiotherapists’ access to personal and medical information is within the remit of standard care and performs the coding for the research data. Researchers outside the Hospital do not have access to personal information and they will receive the data in anonymous (coded) format.

All participants are allocated a subject number upon signing consent. All data collected from that point onwards are defined by subject number only. Data collected are seen by the physiotherapists and used by the research team in coded, anonymised form. Clinical information is incorporated into the final data set by the physiotherapists. Researchers outside the NHS do not have access to any identifiable information. All data are recorded in secure CRFs. Any hard copies and tissue samples (hair) are stored in a locked fire-proof cabinet. Personal data beyond that stored in the NHS system are not collected. Any identifiable and personal data are only accessed by the standard care providers at the Trust. All data are protected and confidentiality maintained following the NHS Code of Confidentiality [139].

The laptop computer used for the computerised testing is encrypted and password protected. The implicit assessment tests are custom made and programmed under Linux operation system with no commercially available programmes on the laptop. The test results are recorded by the unique ID codes. Hair samples are considered as ‘gift’ and kept beyond the scope of this project. It is envisaged that these anonymous samples may be complemented with samples from other healthy and unhealthy populations and used in future projects aiming to assess health. Hair samples collected from living individuals are exempt from the Human Tissue Act [140].

### Research ethics and approval

Owing to the project design and strict inclusion/exclusion criteria, the project does not raise ethical concerns. Written consent is obtained by the physiotherapists after providing full study information. Should a participant decide to withdraw from the study for any reason, the participant and all identifiable data or tissue collected would be removed from the study. Data or hair samples which are not identifiable to the research team may be retained.

The project has no foreseeable risk to the participants and their identity is fully protected. The computerised and self-reported psychological tests are coded by the attending physiotherapist and although they present a cognitive challenge, they are completed in segments between the elements of the physical tests. The physical assessment and the 10-week exercise programme are part of the prescribed treatment. None of the physical tests are to exhaustion.

Involvement in an exercise programme could potentially cause temporary pain, discomfort and/or inconvenience but it is part of the standard treatment and the Trust has a generic risk assessment for gym based exercise. In this study, the risks are minimised by prescribing clinically indicated simple exercises that are suitable for each participant and that each participant is observed doing the exercises under the supervision of a state-registered physiotherapist. Participants may feel inconvenienced at attending pre- and post assessment sessions, however, their time is compensated with gift vouchers. The research design does not have any risk related to its activities.

Testing: computerised testing could have potential acute cognitive fatigue that may affect executive function in an immediate follow-up task that would also require executive control. This has no bearing on normal functioning and should recover within 10 minutes. Although the questionnaire contains some sensitive questions as part of the standard quality of life assessment (e.g. satisfaction with sexual life), they will be analysed collectively at the group level (adherent/non-adherent) and will not be discussed face to face with the participant.

The researchers have no conflict of interest. Psychological testing is added to the exercise programme provided within standard care. Researchers will have no direct benefit or detriment from the outcome of this study. Knowledge generated in this project will serve future studies into treatment adherence, which in turn may lead to increased functional capacity and physical fitness, better heath and improved quality of life. Although there is no direct benefit to participants from the research component (psychological testing), their contribution will lead to a better understanding of motivating factors and barriers to adherence and inform future intervention strategies and support. The level of service provided (standard care) is not affected whether patients take part or not in this study.

Approval for this study was granted by the NHS National Research Ethics Service under reference number 09/H0712/86.

### Collaboration

The project is a collaborative work between two academic institutions, Kingston University and The University of Sheffield; and the *Guy’s and St Thomas’ NHS Foundation Trust* London.

## Discussion

This multidisciplinary project is novel in many ways. The project assesses beliefs about both the underlying goal such as following prescribed treatment; and about the specific behaviours such as exercising or taking the medication, using both implicit and explicit assessments of patients’ beliefs and attitudes. In addition, the project incorporates objective information on adherence to both exercise and medication. The outcome of this study will reveal the extent to which patients adhere to treatment regimens. Psychological assessment results linked to known non-adherence will provide indicators to identify patients who are at risk of non-adherence, and may be used to inform early prevention and intervention programmes.

The reality of recruiting relatively large numbers of eligible participants into this trial has been a challenge, to date lasting 2.5 years. Being solely reliant on appropriate referrals from the wider team initially led to insufficient through flow of appropriate potential participants. At approximately 12 months into the recruitment phase, measures were taken to increase access to suitable candidates though to date, the 40 participants required for the power calculation have not been recruited.

One of the risk elements to the successful research project has been identified was a possibility that by signing up for the study itself has an influence on adherence. In addition to the effort made to obtain data from non-adherent patients to avoid volunteer bias motivation, attendance records of the project participants are continuously monitored and compared to the attendance logs kept since 2002 at the Hospital. Attendance record of the current nonparticipating patients serves as an anchor to compare attendance of the project participants.

Normal business-as-usual gym group clinical activity has been maintained throughout the trial period, however some practical issues ensued which are noteworthy. Firstly self selected attendance rates for trial participants has led to inconsistent group sizes and subsequent unpredictable demand on the therapists involved - particularly when 2 or 3 participants attend for outcome measures during the same session. Secondly, in reality we experienced a large variance in the period of time eligible and consenting participants required to complete the computer and paper-based tests and this was skewed towards longer than expected times. This might be reflective of our cognitive and language inclusion criteria being light in this regard and might inadvertently impacted on drop-out rates and motivation to return for outcome measurement sessions. Lastly we expected that recalling participants for T2 would be problematic with high risk of drop out. We attempted to mitigate it by offering the highest proportion of remuneration at this point of the trial pathway. However despite this T2 outcomes have constituted a number of non-attendees and we will analyse this impact.

**Figure 4:**
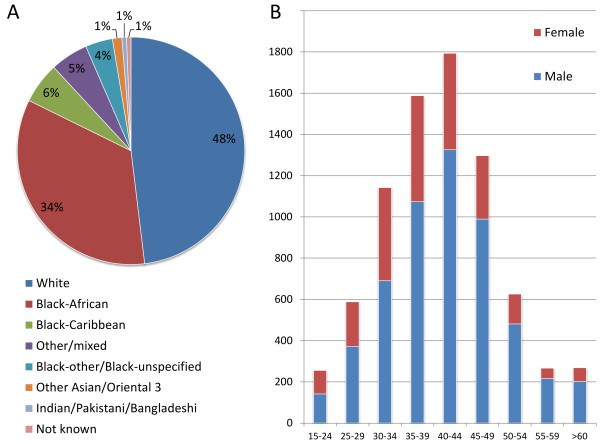
**Local HPA prevalence data for South East London, average of 2008-2010 **[141]. **A:** ethnic distribution. **B:** age and gender distribution.

Our experience so far has shown that inclusion criteria has meant that approximately 1/3 of gym group patients with a need for supervised exercise, did not fulfil inclusion criteria for participation in the trial. While we planned the protocol on real-world patients in reality we could be subjected to typical trial generalisabilty criticism. Published Health Protection Agency (HPA) data implicates that there is a clear link between ethnic background, rate and route of HIV transmission. The majority of people living with HIV in South East London are either white or black African [141] with an increasing number of people over the age of 50. See Figure4 for characteristics of people living with HIV in South East London during the trial period. To date, the modest cohort in this study is reflective of local prevalence data.

Reasons for exclusion to date are language (n = 11), cognition (n = 10), age (n = 7) and cART naivety (n = 2). At the time of writing the mean age of group attendee is 46.5 years (range 19-72 years). Sixteen percent (15/94) of gym group attendees fell above our 55 years cut off for the trial. On reflection, the attending physiotherapists concede that the views of these patients would have been as valuable as their younger peers. Should our study progress to stage 2, we do accept that this should be considered; changing the upper age limit for recruitment would encompass older adults who are living longer due to high quality cART as well as those contracting the virus later in life.

One potential confounding factor that has emerged during data collection is potential mismatch between the medication records on drug treatment regime and what patients may actually take, which could easily lead to categorisation as under-adherent (taking less than expected) or over-adherent (taking more than expected). Over-adherence has been observed in relation to other medications and linked to the number of pills [142,143] and poor cognition [143], although elsewhere over-adherence was observed at a higher rate among those who were on the once-daily regime [144]. A fluctuating pattern of over- and under-adherence to HAART has been documented [145], suggesting that adherence to HAART is not a stable characteristics thus any observation made based on hair analysis is limited to the study period. Longitudinal study following both medical and exercise adherence, along with potential reasons for over-adherence is warranted. Whilst the medical records provide detailed and accurate information on the drug treatment regime prescribed to the patient, including changes over time, these records may not be the exact reflection of what patients may take. There is a possibility of a patient having more than one hospital number in which case there is a temporary mismatch between the expected and detected drug in patients’ hair samples. In summary, caution must be used when making inferences from the observed discrepancies between medical records and behaviour, as deviation from the medical records may not automatically indicate fault on the patient’s side.

Measures to control for a precise matching between the time periods investigated and length of hair sample as possible is advisable. One such measure is using axilla hair and shaving to skin level at baseline to ensure that previous treatment regime does not have influence on the results. This however would be likely to make recruitment more challenging as participants have to accept having a bald patch at the start of the study if they normally have axilla hair, and conversely, having axilla hair temporarily for those who routinely remove it. Nevertheless, it could be a feasible alternative to using scalp hair, axilla hair being less affected by chemical treatment. Should the project progress to stage 2, we envisage to create a shorter testing protocol, retaining only those variables that significantly differentiate between adherent and non-adherent patients. Additional factors may be added if results from this study highlight the need.

## Trial status

Data collection has started in 2009 and is currently ongoing.

## Competing interest

The authors declare that they have no competing interests.

## Authors’ contributions

AP, DN and KH initiated the project. AP and GJ are principle investigators. AP conceived the study design with assistance from PS, DN, GJ and KH. GJ, KH and RM facilitate recruitment and collect the data in the clinical setting. TN developed the software for the computerised psychological tests. AP and PS are responsible for analysing and interpreting the psychological data. DN is responsible for overseeing the chemical analyses. All authors have read and approved the final version of the manuscript.

## Funding

The project received funding from The British Academy SG-52075 and Guy’s & St Thomas’ NHS Foundation Trust Research and Development Salary Support. Project CLRN number: 7842.

## Pre-publication history

The pre-publication history for this paper can be accessed here:

http://www.biomedcentral.com/1471-2458/12/587/prepub

## Supplementary Material

Additional file 1 Variables and measures: Trials_HIV_2012_Additional file 1. Click here for file

Additional file 2 Reasons for non-adherence to medication and exercise: Additional file 2. Click here for file
