# Supplementary material for: Understanding how adherence goals promote adherence behaviours: a repeated measure observational study with HIV seropositive patients
Source: BMC Public Health. 2012 Aug 1;12:587. doi: 10.1186/1471-2458-12-587 (PMC3490813; doi:10.1186/1471-2458-12-587)
Supplement: Additional file 1 — Variables and measures: Trials_HIV_2012_Additional file 1. [file 1471-2458-12-587-S1.pdf]

Additional file 1: Variables and measures

Category	Variable	Test	Test/Reference	S1	T0	T1	T2
**Cognitive ability**	Mental status	ST	Mini Mental State Examination [1,2]	✓			
	English language (level)	SR	Education and self-reported difficulty understanding the screening questionnaire	✓			
**Behavioural predictors**	Attitude, intention, subjective norms and self-efficacy relating to goals (Goal: following doctor's recommendation)	E-SR, I	Modified Brief IAT and matching self-report (Figure 2 and Table 1)		✓	✓	✓
	Attitude, intention, subjective norms and self-efficacy relating to specific behaviours (Implementation: taking medication and doing the prescribed exercise)	E-SR, I	Modified Brief IAT and matching self-report (Figure 2 and Table 1)		✓	✓	✓
	Outcome beliefs regarding medication and exercise	SR	3 items each		✓	✓	✓
	Anticipated regret	SR	1 item each		✓	✓	✓
	Self-efficacy	SR	3 items		✓	✓	✓

**Wellbeing**	Perceived quality of life and health	SR	Single item self-assessment	✓			
	Perceived quality of life	SRS	Functional Assessment of Human Immunodeficiency Virus Infection (FAHI), Version 4 [3]		✓	✓	✓
	Perceived health	SR	Single item self assessment	✓			
**Personality**	Need for cognition	SRS	Rational Experimental Inventory Scale [4]		✓	✓	✓
	Conscientiousness	SRS	NEO FFI subscale [5]		✓	✓	✓
	Social desirability	SRS	Short form M-C 2 (10) [6]		✓	✓	✓
	Global self-esteem	I	single question implicit global self esteem [7]	✓	✓	✓	✓
**Behaviour**	Physical activity involved in daily work	SR	Self-report	✓			
	Physical Activity	SR	International Physical Activity Questionnaire [8]		✓	✓	✓
	Adherence to medication	SR	Direct question		✓	✓	✓
	Reasons for non-adherence to medication	SR	ACTG Baseline Adherence Questionnaire [9]		✓	✓	✓
	Adherence to exercise (intervention)		Attendance log		✓		
	Adherence to exercise (off site)	SRS, SR	Self-report based on [9], Exercise diary		✓	✓	✓

**Physical assessment**	Height	DM	Measure taken by physiotherapist		✓		
	Weight, circumferential measures, rest heart rate	DM	Measure taken by physiotherapist		✓	✓	✓
	Fitness	DM	6 minute walk test [10,11]		✓	✓	✓
	Strength	DM	One repetition maximums for eight exercises		✓	✓	✓
	Flexibility	DM	Sit & reach test [12]		✓	✓	✓
**Clinical data**	Viral load (copies/mL), CD4 counts (cells/mm^3), prescribed medication details, referral reasons, the presence of other chronic illness(es), route of infection, time since diagnosis	MR	Anonymised medical records				✓
**Demographics**	Age, sex and ethnic origin	SR<br>MR	Anonymised medical records	✓			✓
**Hair sample**	50g cut hair	DM	taken by physiotherapist				✓

E: explicit self-report, I: implicit association, DM: direct measure, MR: medical record, SR: self-report SRS: self-reported scale, ST: screening test

References:

1. Folstein MF, Folstein SE, McHugh PR: **"Mini-mental state". A practical method for grading the cognitive state of patients for the clinician.** *J Psychiatric Res* 1975, **12:**189–198.
2. O'Bryant SE, Humphreys JD, Smith GE, Ivnik RJ, Graff-Radford NR, Petersen RC, Lucas JA: **Detecting dementia with the mini-mental state examination in highly educated individuals.** *Arch Neurol* 2008, **65:**963-967.
3. Peterman AH, Cella D, Mo F, McCain N: **Psychometric validation of the revised Functional Assessment of Human Immunodeficiency Virus Infection (FAHI) quality of life instrument.** *Qual Life Res* 1997, **6:**572-584.
4. Epstein S, Pacini R, Denes-Raj V, Heier H: **Individual differences in intuitive–experiential and analytical–rational thinking styles.** *J Pers Social Psychol* 1996, **71:**390-405.
5. Costa PT, McCrae RR: *The NEO Five Factor Inventory (NEO FFI).* Florida: Psychological Assessment Resources Inc. 1992.
6. Strahan R, Gerbasi KC: **Short, homogeneous versions of the Marlowe-Crowne Social Desirability Scale.** *J Clin Psychol* 1972, **28:**191-193.
7. Gebauer JE, Riketta MB, Broemer P, Maio GR: **"How much do you like your name?" An implicit measure of global self-esteem.** *J Exp Soc Psychol* 2008, **44:**1346-1354.
8. International Physical Activity Questionnaire (IPAQ).[https://sites.google.com/site/theipaq/]
9. NAISD Adult AIDS Clinical Trials Group: **ACTG Baseline Adherence Questionnaire.** [https://actgnetwork.org].
10. Guyatt GH, Sullivan MJ, Thompson PJ, Fallen EL, Pugsley SO, Taylor WA, Bernman LB: **The 6-minute walk: a new measure of exercise capacity in patients with chronic heart failure.** *Can Med Assoc J* 1985, **132:**919-923.
11. Passantino A, Lagioia R, Mastropasqua F, Scrutino D: **Short-term change in distance walked in 6 minutes is an indicator of outcome in patients with chronic heart failure in clinical practice.** *J Am Coll Cardiol* 2006, **48:**99-105.
12. Sit & Reach Test [http://www.topendsports.com/testing/tests/sit&reach.htm].
