# Supplementary material for: Understanding how adherence goals promote adherence behaviours: a repeated measure observational study with HIV seropositive patients
Source: BMC Public Health. 2012 Aug 1;12:587. doi: 10.1186/1471-2458-12-587 (PMC3490813; doi:10.1186/1471-2458-12-587)
Supplement: Additional file 2 — Reasons for non-adherence to medication and exercise: Additional file 2. [file 1471-2458-12-587-S2.pdf]

Additional file 2: Reasons for non-adherence to medication and exercise

Medication specific	Both	Exercise specific
In the past 3 months how often have you missed taking your medication / attending your exercise session because you…		
had too many pills to take	were away from home	felt tired
wanted to avoid side effects	were busy with other things	don't like the gym
did not want others to notice you are taking medication	simply forgot	felt embarrassed if other people saw you exercising
felt like the drug was toxic/harmful	had a change in daily routine	don't feel comfortable in exercise clothes
fell asleep/slept through dose time	felt depressed/overwhelmed	didn't like being ridiculed by others for exercising
had problems taking pills at specified times (with meals, empty stomach, etc.)	felt sick or ill	felt that people important to you complained about the time commitment
ran out of pills	felt good	felt like exercise was painful
felt taking medication pointless		were injured
felt taking medication all the time tiresome		felt that you were out of your own environment
		had personal problems
		disagree with the reason(s) why exercise was prescribed to you
		felt physical discomfort during exercise
		felt doing exercise was pointless
		felt that exercise twice a week tiresome
